# Decreased precipitation reduced the complexity and stability of bacterial co-occurrence patterns in a semiarid grassland

**DOI:** 10.3389/fmicb.2022.1031496

**Published:** 2022-12-22

**Authors:** Jinlong Wang, Chunjuan Wang, Jinwei Zhang, Xuefeng Wu, Yu Hou, Guiyun Zhao, Haiming Sun

**Affiliations:** ^1^Traditional Chinese Medicine Biotechnology Innovation Center in Jilin Province, College of Science, Beihua University, Jilin City, China; ^2^Department of Grassland Science, College of Animal Science and Technology, Northeast Agricultural University, Harbin, China; ^3^Chongqing Institute of Quality and Standardization, Chongqing, China

**Keywords:** co-occurrence networks, soil bacterial community, precipitation gradients, semiarid grasslands, drought

## Abstract

**Introduction:**

Grasslands harbor complex bacterial communities, whose dynamic interactions are considered critical for organic matter and nutrient cycling. However, less is known about how changes in precipitation impact bacterial interactions.

**Methods:**

We conducted precipitation manipulation experiments in the Eastern Eurasian Steppe in China and constructed co-occurrence networks for bacterial communities.

**Results:**

The network topological features of the bacterial communities exhibited considerable differences among increased precipitation, control, and decreased precipitation gradients. The bacterial co-occurrence pattern in the increased precipitation gradient was the most complex and stable, with a large network size, followed by those of the control and decreased precipitation gradients. Soil moisture (SM) was the primary factor influencing the complexity, size, and stability of bacterial networks across different precipitation gradients, followed by total nitrogen (TN), belowground biomass, aboveground biomass, and total carbon (TC).

**Discussion:**

Our results indicate that drought conditions reduce the complexity and stability of the bacterial community, and future changes in precipitation will greatly reshape bacterial interactions in semiarid grasslands. Overall, these findings could enhance our understanding of how microbes respond to changing precipitation patterns by regulating their interactions in water-limited ecosystems and will improve our ability to predict the impacts of precipitation regime change on ecosystem nutrient cycling and feedback between ecosystem processes and global climate change.

## 1 Introduction

Climate change refers to any shift in climate over an extended period, either caused by natural variability or human activity (Sun et al., 2013). Global climate change has already significantly affected soil ecosystems through changes in temperature, precipitation patterns, nitrogen fertilization, and CO_2_ emissions (Wang et al., 2018). Global and local precipitation patterns, as important components of the global water cycle, are changing and will continue to do so (Wang et al., 2021). Soil microorganisms are important components of grassland ecosystems. Due to their important roles in regulating organic matter, nutrient cycling, and promoting energy flow, microorganisms are crucial for maintaining soil fertility and sustainability (Fierer, 2017; Jiang et al., 2021). In addition, microorganisms play a wide range of biochemical roles; they serve as important storage sites and sources of nutrients in the soil. Therefore, microorganisms are highly sensitive to changes in the habitat environment (Nielsen et al., 2002), and the composition of soil microorganisms varies temporally, geographically, and spatially (Chen et al., 2017, 2021).

Microbes are highly sensitive to changes in soil water availability and physicochemical conditions in semiarid grasslands (Chen et al., 2019; Yang et al., 2021). In water-limited ecosystems such as arid and semiarid grasslands, water availability is considered one of the most critical factors affecting soil microbial activity and nutrient cycling (Wang et al., 2018). The response of soil bacterial communities to changes in precipitation has been extensively studied over the recent few decades. Soil moisture (SM) is believed to be one of the major constraints affecting microbial diversity, community structure, and activity in arid ecosystems (Li et al., 2016). The variation in precipitation can affect soil bacterial communities directly by changing soil water availability and indirectly by altering soil nutrient availability and plant community composition and productivity (Wu et al., 2020). When shifts in soil microbial communities occurred in response to variations in precipitation, soil water content, pH, and soil nutrient status, such as total phosphorus (TP) and total nitrogen (TN) concentration, were the most critical drivers (Wu et al., 2020; Yang et al., 2021). Microbes coexist in complex arrangements, and interspecies relationships play a crucial role in community assembly and ecosystem function (Hallam and McCutcheon, 2015; Shi et al., 2016). Therefore, identifying and characterizing the interactions that occur among soil microorganisms are vital for understanding microbial community structure and diversity, as well as ecosystem function (Shi et al., 2016). Although extensive studies have been conducted to examine the influence of soil water availability on microbial community composition, diversity, and activity in semiarid grasslands (Koyama et al., 2018; Qin et al., 2020; Yang et al., 2021), the effects of changes in precipitation on belowground microbial community co-occurrence patterns are still not fully understood.

The formation of a microbial community depends on the relative balance of community assembly processes (Stegen et al., 2015). Therefore, selection is an important ecological process driving the assembly of microbial community structures and interactions between microbial species, as a selective force, contribute to the assembly of microbial communities (Hunt and Ward, 2015) and biogeochemical cycling (Morriën et al., 2017). Currently, an increasing number of microorganism network structures in different environments are being revealed (Wu et al., 2021; Yun et al., 2021; Zhou et al., 2021). Microbial network analysis has been used to explore complex microbial assemblages in the environment (Shi et al., 2016). Compared to the traditional alpha diversity and beta diversity metrics, network analysis provides new insights into the structure of microbial communities (Wang et al., 2018). Mounting evidence indicates that the properties of ecological networks, which reflect interactions among coexisting organisms, affect the reactions of communities to environmental variations, including climate extremes (Wagg et al., 2019). These network investigations offer insights beyond those of simple abundance richness and composition metrics and serve as a new methodological tool for the in-depth analysis of microbial community ecology (Shi et al., 2016). However, interactions among the members of microbial assemblages, microbiome complexity, and stability within different precipitation gradients remain poorly understood. Therefore, understanding the responses of bacterial co-occurrence patterns to variations in precipitation is imperative for predicting the effects of precipitation regime changes on the organization and dynamics of microbial interactions and niches, as well as biogeochemical cycling.

To better understand the effects of variations in precipitation on microbial interactions and community assembly in semiarid grasslands, we conducted a 4-year precipitation manipulation experiment in a semiarid grassland in northern China. We used Gephi 0.9.2 and R to construct integrated co-occurrence networks of bacterial community datasets from 54 samples (collected in 2016, 2018, and 2019) associated with different precipitation levels (increased precipitation, decreased precipitation, and control). We hypothesized that (i) the co-occurrence patterns of bacteria would be more complex and stable when precipitation increased and (ii) soil water availability and soil nutrient status are the main factors that modulate microbial co-occurrence patterns in response to precipitation. Our study provides a novel experimental perspective on drought conditions that reduce the complexity and stability of bacterial communities.

## 2 Materials and methods

### 2.1 Study area and experimental design

Precipitation manipulation experiments were performed at the Songnen Grassland Ecological Research Station of Northeast Normal University, Chang Ling County, in Jilin Province, China (44°45′N, 123°45′E), located on the Eastern Eurasian Steppe. The region is characterized by dry, cold winters and relatively humid, warm summers, with a typical mid-temperate monsoon climate. The average annual temperature and annual precipitation range from 4.6 to 6.4°C and 280 to 400 mm, respectively. Approximately 80% of the precipitation events over the past five decades (1961–2010) occurred from June to August.

From 2016 to 2019, rain manipulation experiments were conducted annually from 1 May to 1 October in a topographically uniform area. The following three precipitation treatments were tested: control (0%), 30% decreased precipitation (–30%), and 30% increased precipitation (+30%). A total of six replicates for each treatment [18 plots (3.5 m × 3.5 m) in total] were established. The treatments were randomly distributed across the 18 plots. We installed polyvinyl chloride (PVC) plates at a depth of 50 cm in the soil column surrounding each plot to isolate each plot hydrologically. Each plot included a 0.5 m external buffer to allow access to the plot and to minimize the edge effect associated with the infrastructure.

We conducted vegetation sampling (one 1 m × 0.1 m quadrat in each plot) in mid-September of 2016, 2018, and 2019. The aboveground living plants were harvested to measure plant biomass. Root samples were obtained with a root drill (10 cm diameter, 30 cm depth). Two root cores were collected for each treatment and cleaned on the same day by removing soil particles under running water, and the belowground biomass was subsequently measured. Soil samples were collected simultaneously; five soil cores (0–30 cm depth) were collected from each plot. Subsequently, the samples were transported to the laboratory in a refrigerated box and stored at −20°C. In total, we obtained 54 soil samples (three treatments × six replicates × 3 years). Each sample was divided into three parts. The first part was maintained at −20°C for DNA extraction. The second part was used to measure SM; the soil was dried at 105°C for 48 h and then weighed. The third part was used to test the physical and chemical parameters of soil.

### 2.2 Physical and chemical analysis of soil

Soil pH and electrical conductivity were determined in a 1:5 (v v^–1^) soil:water suspension using a pH meter and an electrical conductivity meter (INESA Instrument, Shanghai, China), respectively. Soil total carbon (TC) and TN were determined using an elemental analyzer (vario EL cube, Elementar, Langenselbold, Germany). TP was determined using the molybdenum blue ascorbic acid method after H_2_SO_4_–H_2_O_2_ digestion.

### 2.3 DNA extraction and polymerase chain reaction

Genomic DNA was extracted from 0.5 g of soil using the Power Soil DNA Kit (Menlo Park, CA, USA). The V4 region of bacterial 16S was subjected to PCR using the following primer pair: 515F, 5′-GTG CCA GCM GCC GCG GTA A-3′ and 806R, 5′-GGA CTA CHV GGG TWT CTA AT-3′. After quantification, amplicons from the different sites were pooled at equimolar concentrations. Sequencing was performed using Illumina MiSeq, according to the standard protocols of Majorbio BioPharm Technology Co., Ltd., (Shanghai, China). The details of DNA extraction, quality control, and raw data processing were provided by Wang et al. (2015).

### 2.4 Network analysis

Co-occurrence network analysis was used to explore the interactions among soil bacterial species at different precipitation gradients. Co-occurrence network analysis of bacterial communities was conducted at the operational taxonomic units (OTU) level. The Spearman correlation method was used to assess correlations between bacterial species. Values of r > |0.8| and *P* < 0.01 were used as thresholds for the construction of the co-occurrence network. Correlation matrix analysis was performed using the “Hmisc” package in R software, and sub-network properties were analyzed using the “igraph” package. Network visualization was performed using Gephi version 0.9.2. Network stability was assessed by removing nodes from the static network to evaluate the rate of robustness degradation, and network robustness was assessed using natural connectivity (Peng and Wu, 2016). Random forest analysis *via* the “randomForest” function in R was used to analyze the relative importance of the physicochemical parameters and plant aboveground and belowground biomass in the bacterial network structure. The importance of variables was determined by the value of %IncMSE (increase in mean squared error), calculated by the “importance” function.

The nodes of each network were classified into the following four categories: peripherals (Zi < 2.5, Pi < 0.62), network hubs (Zi ≥ 2.5, Pi ≥ 0.62), module hubs (Zi ≥ 2.5, Pi < 0.62), and connectors (Zi < 2.5, Pi ≥ 0.62) by within-module connectivity (Zi) and among-module connectivity (Olesen et al., 2007). Based on their important roles in network topology, network hubs, module hubs, and connectors are considered potential keystone taxa (Banerjee et al., 2016).

## 3 Results

### 3.1 Overall sequencing information

After quality control, a total of 3,940,166 sequences ranging from 201 to 540 bp in length (the length of most sequences was 431 bp) were obtained from 54 samples *via* Illumina MiSeq sequencing. Based on 97% similarity, 1,885 OTUs were detected. These OTUs belonged to 28 phyla, 73 classes, 170 orders, 255 families, 444 genera, and 801 species. The majority of the bacterial sequences belonged to the phyla *Actinobacteria* (relative abundance of 39.6%), *Proteobacteria* (21.0%), *Chloroflexi* (13.5%), *Acidobacteria* (12.3%), *Gemmatimonadota* (2.4%), and *Firmicutes* (2.3%). The species accumulation curves (Supplementary Figure 1) tended to reach a saturation plateau with increasing sample number, which indicated that the number of bacterial sequences obtained represented the bacterial communities well. Our results demonstrated that the alpha diversity and beta diversity of bacterial communities did not vary significantly among the control, increased precipitation, and decreased precipitation gradients in any experimental year (Supplementary Figures 2, 3 and Supplementary Tables 1, 2).

### 3.2 Topological properties of bacterial networks at different precipitation levels

**FIGURE 1:**
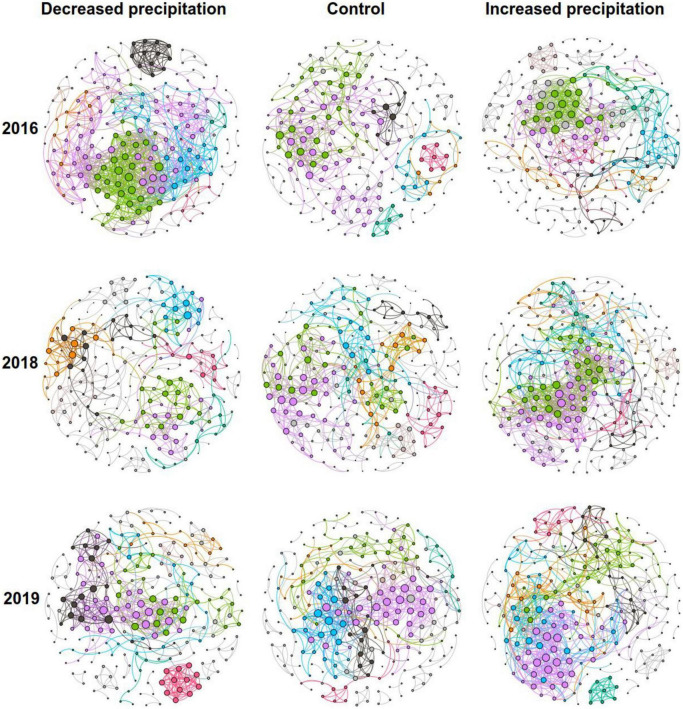
Co-occurrence network patterns of communities of constructed bacteria in different precipitation gradients. The six largest modules are represented by different colors, and the other modules are represented in gray.

**FIGURE 2:**
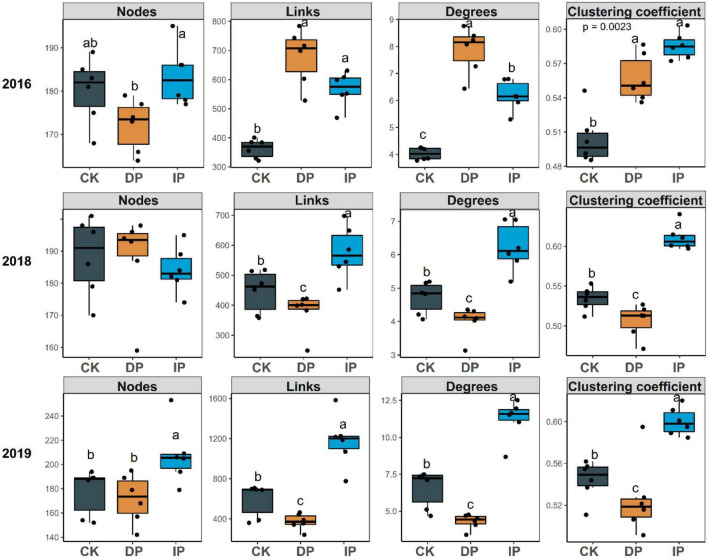
Topological properties of the bacterial co-occurrence networks in different precipitation gradients in 2016, 2018, and 2019. CK, control; DP, decreased precipitation; IP, increased precipitation. Different lowercase letters indicate significant differences among the three sites (*P* < 0.05).

To determine the general effects of precipitation on bacterial interactions, nine bacterial networks for the control, decreased precipitation, and increased precipitation gradients in 2016, 2018, and 2019 were constructed individually (Figure 1), and their sub-network topological parameters (Figure 2) were measured. In 2016, except for the number of nodes associated with decreased precipitation, the sub-networks for increased and decreased precipitation contained higher links, degrees, and clustering coefficients than the control (Figure 2). However, in 2019, there were significant differences in the number of nodes, and in 2018 and 2019, there were significant differences in the number of edges, average degree, density, and clustering coefficient, in the order of increased precipitation > control > decreased precipitation (Figure 2). These results demonstrate that in the 1-year experiment analyzing the effects of a short-term disturbance, both decreased and increased precipitation resulted in a more compact and aggregated bacterial co-occurrence pattern; the increased precipitation and decreased precipitation networks were larger and contained more nodes and links than the control network. However, in the 3-and 4-years experiments analyzing the effects of a long-term disturbance, the bacterial interaction pattern associated with increased precipitation was more compact and aggregated than those associated with the control and decreased precipitation. Bacteria formed larger networks with more nodes and links in the following order: increased precipitation > control > decreased precipitation.

**FIGURE 3:**
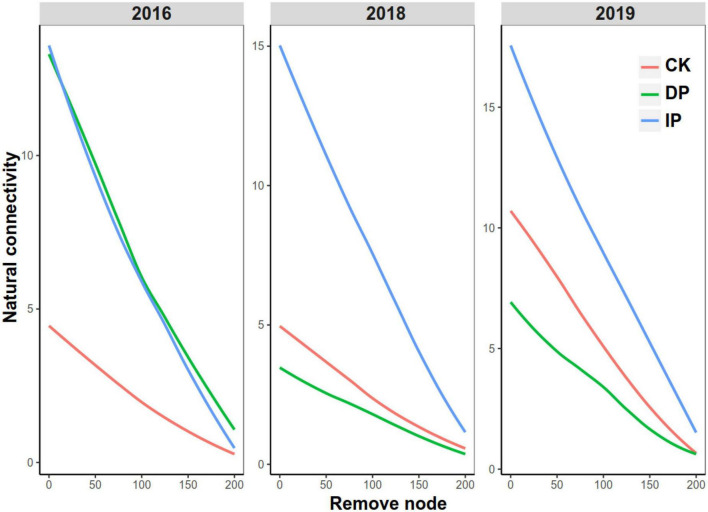
Natural connectivity of bacterial networks in different precipitation gradients in 2016, 2018, and 2019. CK, control; DP, decreased precipitation; IP, increased precipitation.

Finally, we conducted a natural connectivity analysis to test the robustness of the bacteria in response to different precipitation gradients in different experimental years. In 2016, the natural connectivity values of the networks in the increased and decreased precipitation gradients were higher than that in the control (Figure 3). In 2018 and 2019, the increased precipitation network had the highest natural connectivity value, followed by the control and decreased precipitation networks (Figure 3). These results indicate that higher precipitation strengthened bacterial stability at our study sites. Subsequently, the natural connectivity was recalculated after the nodes were removed. The natural connectivity of the increased precipitation, control, and decreased precipitation networks dropped sharply when the nodes were removed.

### 3.3 Module hubs and connectors as putative keystone taxa

Bacterial co-occurrence patterns differed significantly among the control, decreased precipitation, and increased precipitation networks. The networks became more complex, larger, and more clustered as precipitation increased (Figures 1, 2). Nine phyla, namely, *Actinobacteria*, *Acidobacteria*, *Chloroflexi*, *Proteobacteria*, *Gemmatimonadetes*, *Myxococcota*, *Firmicutes*, *Cyanobacteria*, and *Planctomycetes*, had relative abundances greater than 1%. The relative abundances of different phyla involved in the network (Supplementary Table 3) varied among the different precipitation levels in each experimental year.

**FIGURE 4:**
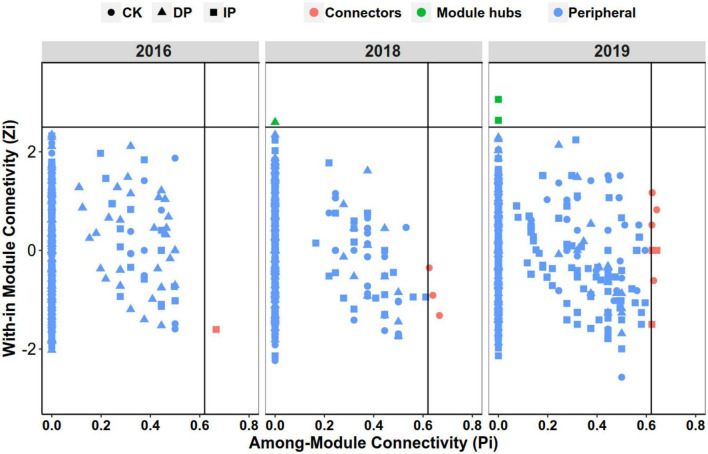
Identification of keystone taxa based on their topological roles in different precipitation gradient networks in 2016, 2018, and 2019. Module hubs are identified as Zi ≥ 2.5, Pi < 0.62; connectors are identified as Zi < 2.5, Pi ≥ 0.62. CK, control; DP, decreased precipitation; IP, increased precipitation.

Within-module connectivity (Zi) and among-module connectivity (Pi) values were used to determine the roles of each node in the network. Each node was classified as a peripheral, connector, module hub, or network hub. No network hubs were detected. A large proportion of the nodes were classified as peripherals, suggesting that the majority of the nodes had only a few links, mostly to nodes within their own modules. In all nine bacterial networks, peripherals accounted for > 99% of the total nodes; connectors, 0.6%; and module hubs, 0.2%. The taxonomic composition of keystone taxa is shown in Figure 4 and Supplementary Table 4. In 2016, only one node in the three networks was classified as a connector, belonging to *Chloroflexi*. In 2018, one module hub belonged to *Firmicutes* in the decreased precipitation network, and three connectors in the control originated from *Gemmatimonadota*, *Chloroflexi*, and *Actinobacteria*, respectively. In 2019, two module hubs in the increased precipitation network belonged to *Chloroflexi* and *Myxococcota*. In the control, five connectors belonged to *Actinobacteriota*, and another five belonged to *Actinobacteria* (2), *Chloroflexi* (2), and *Proteobacteria* (1).

### 3.4 Linking network-level topological features to soil and plant properties

In 2016, there were no significant differences in soil pH, electrical conductivity, TN, TC, or TP among precipitation levels, whereas SM and aboveground biomass increased significantly with increased precipitation (Supplementary Table 5). However, TN, TC, SM, and aboveground biomass exhibited remarkable differences in 2018, and almost all the factors exhibited remarkable differences in 2019 (Supplementary Table 5). The TC, TN, and TP associated with increased precipitation were higher than those associated with the control and decreased precipitation.

**FIGURE 5:**
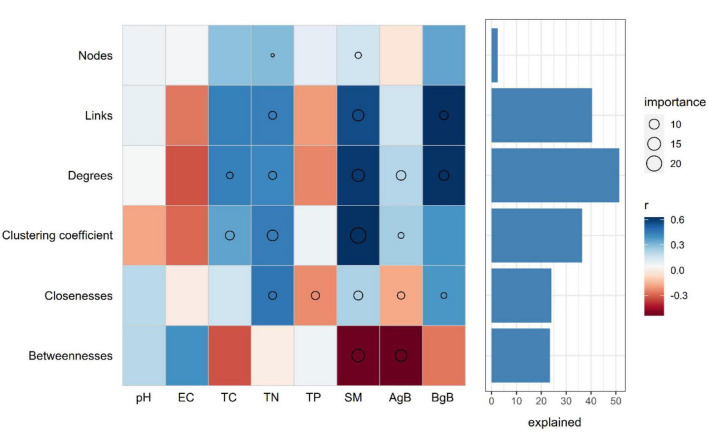
Potential contributions of the soil properties and plant aboveground and belowground biomass to the dissimilarities of network topological features. Circle (*P* < 0.05) size represents variable importance (i.e., the proportion of explained variation calculated *via* multiple regression modeling and variance decomposition analysis). Colors represent Spearman correlations. EC, elecstrical conductivity; TC, soil total carbon; TN, soil total nitrogen; TP, soil total phosphorus; SM, soil moisture; AgB, aboveground biomass; BgB, belowground biomass.

We further evaluated the relative contribution of soil physicochemical parameters to the topological features of networks for different precipitation gradients. Random forest analysis was performed to identify correlations between the network topological features and soil and plant factors. SM was the primary factor influencing the topological features of the bacterial networks. TC, TN, TP, aboveground biomass, and belowground biomass were also important environmental attributes controlling the soil bacterial network structure (Figure 5). Link number, average degree, and clustering coefficient were positively correlated with SM, belowground biomass, and TN. SM and aboveground biomass were negatively correlated with betweenness (Figure 5).

## 4 Discussion

Changes in precipitation and soil water availability, caused by either anthropogenic simulation experiments or natural precipitation gradients, have significant effects on microbial alpha and beta diversity (Ren et al., 2017; Shi et al., 2020; Yang et al., 2021). Microorganisms in the soil are linked through robust ecological interactions, and changes in these interactions may cause changes in ecosystem function (Zhou et al., 2021). However, few studies have been conducted on the microbial interactions that occur in response to changes in precipitation. Therefore, we investigated the effect of precipitation manipulation on bacterial interactions, to complement the traditional measurements of alpha and beta diversity.

We found that the co-occurrence patterns and topological features of the bacterial network structure varied significantly among the control, increased precipitation, and decreased precipitation treatment groups (Figures 1, 2). This result supported our first hypothesis. Interestingly, in 2016, the first experimental year, both increased and decreased precipitation were associated with larger and more complex networks than the control, whereas in 2018 and 2019, the size and complexity of the networks increased with the increase in precipitation. These results are consistent with those reported by Wang et al. (2018), who found that microbial networks become more complex as precipitation increases in natural precipitation gradients. In the 1-year experiment in 2016, both increased and decreased precipitation increased the complexity and size of bacterial networks, most likely because some bacterial taxa are sensitive to short-term changes in precipitation and water availability (de Vries et al., 2018). This sensitivity produces an increase in bacterial network complexity and size, which increases the resistance of the bacterial communities to adverse environmental conditions (Zhao et al., 2019). However, long-term precipitation changes (3–4 years) produced bacterial adaptations; eventually, the size and complexity of the networks corresponded to changes in precipitation. Altered precipitation influenced not only the bacterial network size and complexity but also the robustness of bacterial co-occurrence relationships. Our results demonstrated that the network of bacteria in increased precipitation gradient had the highest natural connectivity, followed by those in control and decreased precipitation gradient. This is consistent with the trends in network size and complexity. Increased complexity of community interactions leads to increased community stability (Mougi and Kondoh, 2012), and competition can also increase stability (Ghoul and Mitri, 2016).

In this study, we found that the number of key taxa has a consistent trend with the complexity and stability of the network, except for increased precipitation in 2018. Similarly, Liu et al. (2022) also found that the number of keystone taxa had a similar trend with network complexity and stability in lake ecosystems. Keystone species strongly influence other members of the community and play an important role in maintaining ecosystem functions (Banerjee et al., 2018; Li et al., 2020). Networks with more keystone taxa are generally more stable (Liu et al., 2022). Keystone taxa make important contributions to the stability of microbial communities because of their strong and unique biological interactions, and their disappearance may lead to the collapse of modules and networks (Coux et al., 2016; Banerjee et al., 2018; Liu et al., 2022). In addition, keystone taxa are highly connected with a high mean degree, high closeness centrality, and low betweenness centrality scores (Berry and Widder, 2014); these stronger interspecific interactions also play important roles in maintaining the ecological community stability (Mougi and Kondoh, 2012; Qian and Akçay, 2020). In this study, the key taxa were specific to different precipitation levels, due to environmental filtering and associated interspecific interactions, which suggests that the conditions present were not identical over precipitation gradient and supports the context dependency theory that keystone species play critical roles only under certain conditions (Power et al., 1996; Shi et al., 2016). Previous studies have shown that the putative keystone species changed with the changes in the conditions (Lu et al., 2013; Lupatini et al., 2014). Alternatively, functional redundancy may explain the unique keystone taxa detected in the different networks (Shi et al., 2016); that is, different species may play the same functional role over precipitation gradient in different modules.

We found that SM was the most important driver of the bacterial co-occurrence patterns across different precipitation gradients (Figure 5). Previous studies have shown that changes in precipitation can directly affect soil bacterial communities by altering soil water availability (Bagayoko et al., 2000), most likely because some taxa are sensitive to changes in water availability (de Vries et al., 2018). The transformation of bacterial interactions may be due to variations in the ability of different species to adapt to changes in soil water availability. In addition, many studies have shown that drought can lead to greater bacterial isolation, thus reducing competition among bacterial species (Treves et al., 2003; Shi et al., 2020), which in turn decreases the complexity and size of the bacterial network. Higher precipitation can increase SM, soil nutrient availability, and plant productivity, all of which facilitate the formation of more stable network structures, in which microorganisms perform specific ecosystem functions. In addition, a compact, aggregated network structure and strong links between competitors can increase the efficiency of resource transfer, compared to that of isolated competitors (Morriën et al., 2017). Therefore, the small network structure and extremely sparse competitive connections associated with decreased precipitation may have negative effects on biogeochemical functions, and microbial communities may become unstable and vulnerable when disturbances occur. Research has proven that a more compact network structure and stronger interspecific interactions between competitors could increase the efficiency of resource transfer compared with those inhabiting isolated spaces (Morriën et al., 2017). In addition, under drought conditions, the dominant species would outcompete less dominant species, potentially weakening the robustness of soil microbial co-occurrence relationships (Shi et al., 2020).

In addition to SM, we found that TC, TN, and aboveground and belowground biomass also played critical roles in determining the complexity and size of bacterial networks in response to variations in precipitation. This is consistent with previous findings that changes in precipitation can indirectly affect soil bacterial communities by altering soil nutrient availability (Xu et al., 2014) and plant community composition and productivity (Gray et al., 2011; Wu et al., 2020). A decrease in SM can reduce the availability of nutrients in the soil. Under poor nutrient conditions, dominant species become more abundant and outcompete non-dominant species, which may diminish the complexity and size of co-occurring relationships among soil microorganisms (Shi et al., 2020). In contrast, higher levels of precipitation can increase soil nutrient availability, providing favorable conditions for microorganisms to form larger and more complex network structures (Wang et al., 2018). Increased precipitation can also alleviate nutrient diffusion limitations in dry environments, leading to high levels of plant diversity and biomass (Bai et al., 2008). Higher biomass results in more litter decomposition and root exudation, which, in turn, increases soil organic carbon content. Therefore, increasing plant diversity and biomass can increase soil humus concentration, improve nutrient availability, and provide better conditions for microbial growth (Hacker et al., 2015; Lange et al., 2015; Li et al., 2017), ultimately increasing the complexity and scale of microbial networks.

In conclusion, this study demonstrated that the network topological features of bacterial communities exhibited considerable dissimilarities among increased, control, and decreased precipitation gradients. The bacterial co-occurrence pattern associated with increased precipitation was the most complex and stable, with a large network size, followed by those associated with the control and decreased precipitation gradients. SM was the primary factor affecting the complexity, size, and stability of the bacterial networks across different precipitation gradients, followed by TN, TC, and belowground and aboveground biomass. Our results demonstrated that bacterial network structures become more stable and complex in response to increased precipitation, and this phenomenon may affect the ecosystem functions of grassland soils. These findings improve our understanding of how changes in precipitation affect bacterial interactions in semiarid grassland ecosystems and will increase our ability to predict the influence of precipitation changes on the ecosystem nutrient cycling and the ability to feedback between the ecosystem process and global climate change.

## Data availability statement

The original contributions presented in this study are included in the article/Supplementary material, further inquiries can be directed to the corresponding authors.

## Author contributions

JW, CW, and XW proposed and organized the overall project. JZ and CW performed the majority of experiments. JW, GZ, and YH assisted with the laboratory work and laboratory analyses. JW and HS wrote the main text of this manuscript. XW, CW, JZ, and YH contributed to the discussions. JW, HS, and XW contributed to the interpretation of the results and reviewed and edited the final manuscript. All authors have reviewed the manuscript, contributed to the article and approved the submitted version.
