# Supplementary material for: Decreased precipitation reduced the complexity and stability of bacterial co-occurrence patterns in a semiarid grassland
Source: Front Microbiol. 2022 Dec 22;13:1031496. doi: 10.3389/fmicb.2022.1031496 (PMC9815162; doi:10.3389/fmicb.2022.1031496)
Supplement: Supplementary file 1 [file Data_Sheet_1.docx]

Short title [Precipitation and bacterial co-occurrence network]

**Decreased** **precipitation reduced the complexity and stability of bacterial** **co-occurrence patterns in** **a semiarid grassland**

Jinlong Wang^a^, Chunjuan Wang^a^, Jinwei Zhang^b,^*, Xuefeng Wu^c^, Yu Hou^a^, Guiyun Zhao^a^ , Haiming Sun^a,*^

^a^ College of Science, Traditional Chinese Medicine Biotechnology Innovation Center in Jilin Province, Beihua University, Jilin 132013, China

^b^Department of Grassland Science, College of Animal Science and Technology, Northeast Agricultural University, Harbin 150030, China

^c^ Institute of Grassland Science, Key Laboratory of Vegetation Ecology of the Ministry of Education, Jilin Songnen Grassland Ecosystem National Observation and Research Station, Northeast Normal University, Changchun 130024, China

*Correspondence: Jinwei Zhang, zhangjw133@nenu.edu.cn; Haiming Sun, sunhaiming@beihua.edu.cn


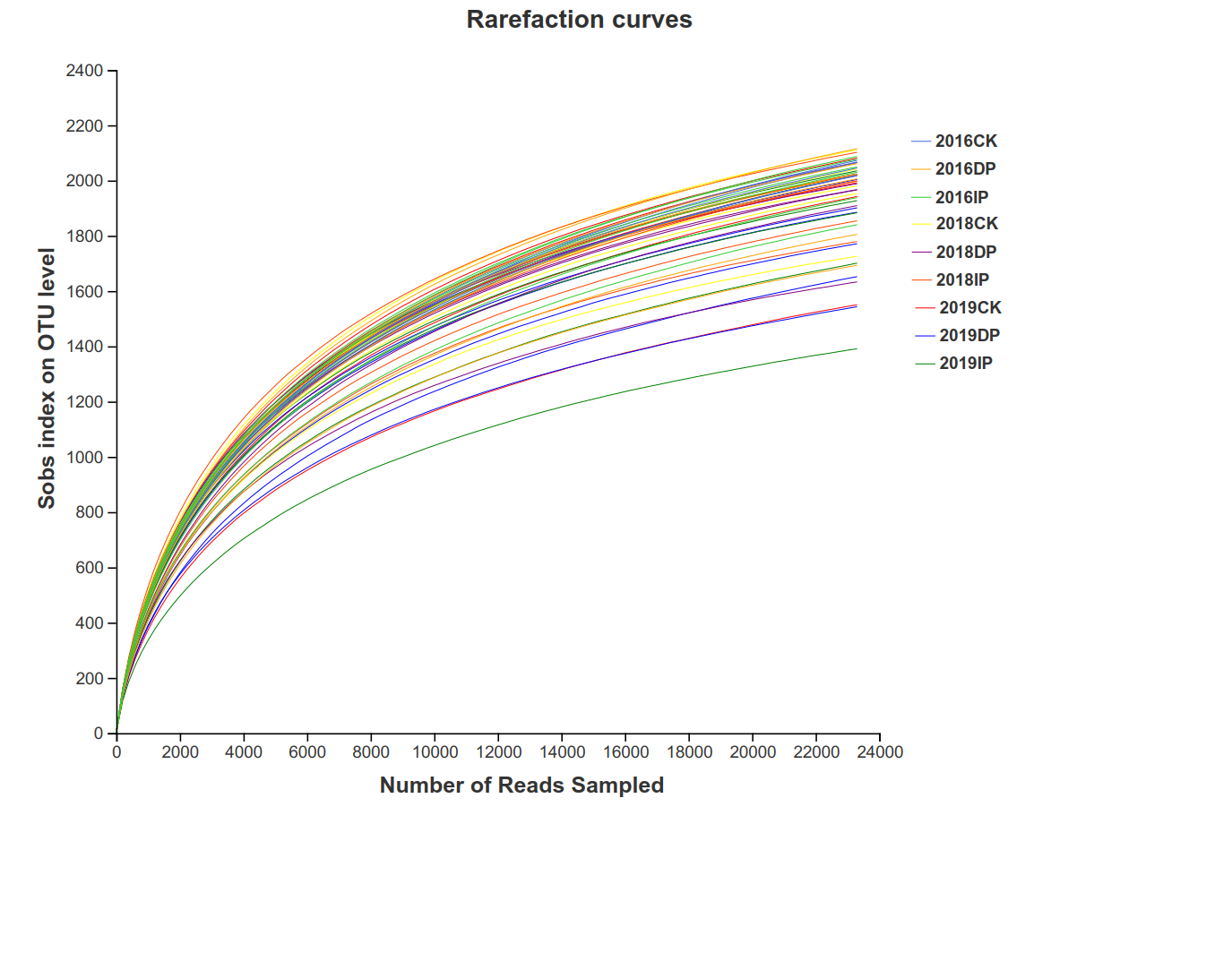


**Figure S1.** Taxa accumulation curves of bacteria in different precipitation gradient in 2016, 2018 and 2019. CK: control; DP: decreased precipitation; IP: increased precipitation.


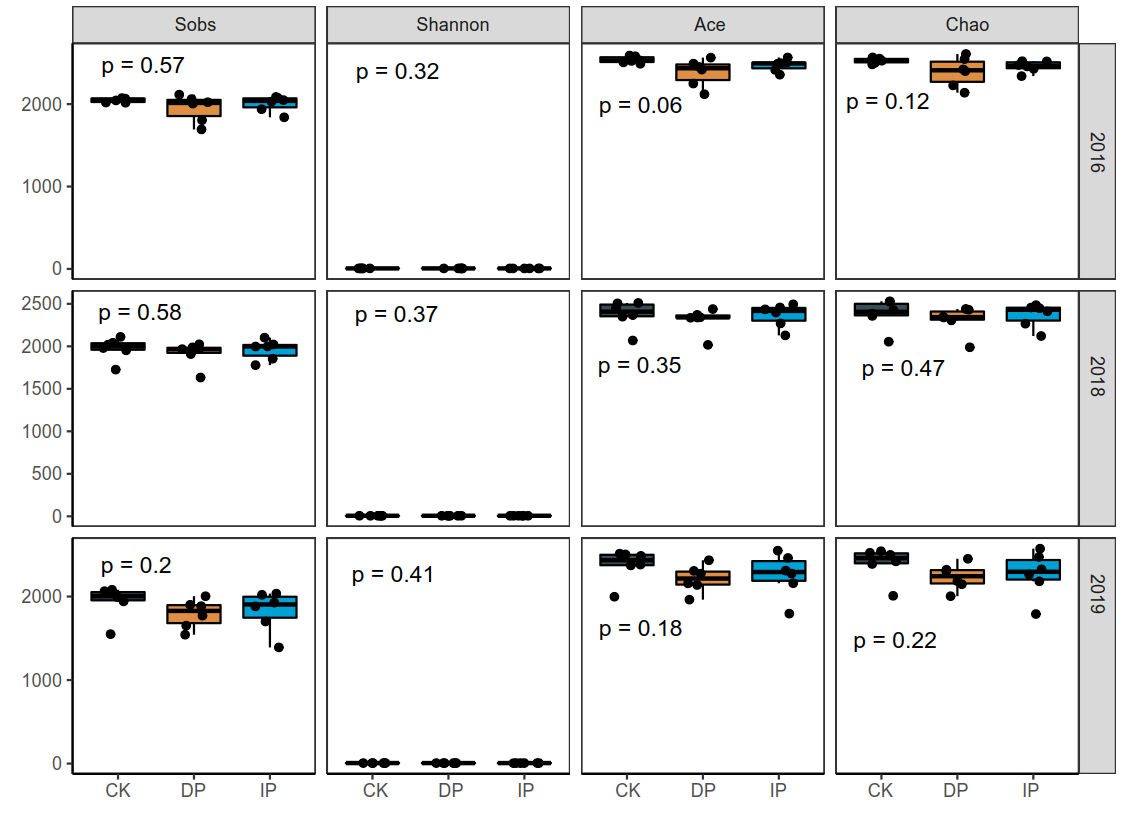


**Figure S2.** The Sobs, Shannon Ace and Chao indices of bacterial in control, decreased precipitation and increased precipitation gradient. CK: control; DP: decreased precipitation; IP: increased precipitation.


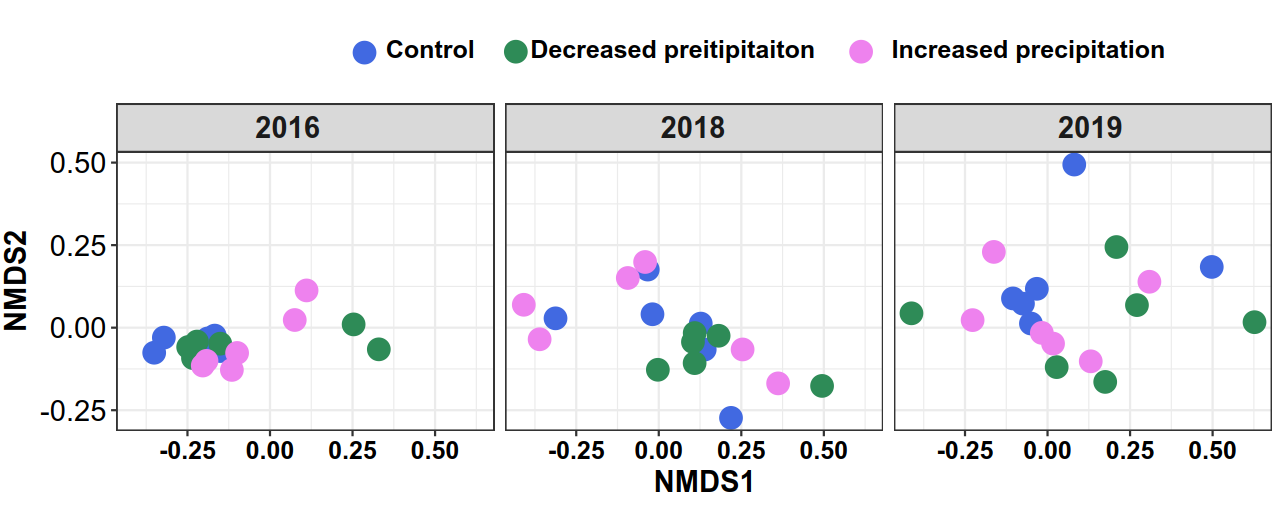


**Figure S3.** Nonmetric multidimensional scaling (NMDS) ordination plot of bacterial communities between control, decreased precipitation and increased precipitation gradient.

| Grassland |  | Shannon | Chao | Ace | Sobs |
| --- | --- | --- | --- | --- | --- |
| 2016 | CK | 6.37±0.02 | 2527.7±13.02 | 2537.06±16.38 | 2047.67±10.14 |
|  | RI | 6.32±0.05 | 2456.57±27.5 | 2471.95±30.14 | 2003.5±39.23 |
|  | RD | 6.20±0.08 | 2390.25±73.74 | 2382.58±67.66 | 1950.5±67.1 |
| 2018 | CK | 6.38±0.06 | 2379.71±71.21 | 2374.83±67.17 | 1972.17±54.03 |
|  | RI | 6.36±0.09 | 2364.92±58.02 | 2363.95±56.59 | 1959±48.64 |
|  | RD | 6.29±0.07 | 2308.48±67.51 | 2307.09±60.1 | 1914.5±58.33 |
| 2019 | CK | 6.31±0.12 | 2397.54±81.4 | 6.31±0.12 | 1941.33±80.91 |
|  | RI | 6.17±0.15 | 2268.78±111.71 | 2258.84±108.53 | 2235.17±63.86 |
|  | RD | 6.13±0.13 | 2235.17±63.86 | 2212.31±66.57 | 1792.67±70.12 |
|  |  |  |  |  |  |
|  | Year | F=1.73 | F=4.11* | F=5.74** | F=4.14* |
|  | Precipitation | F=1.68 | F=2.48 | F=2.82 | F=1.92 |
|  | Interaction | F=0.13 | F=0.20 | F=0.23 | F=0.20 |

**Table S1.** Estimators of bacterial diversity in different precipitation levels.

Estimators were calculated for each replicate samples. Sobs is the observed number of OTUs. Results from linear mixed model (LMM) are presented as F- and P value (* means p < 0.05, ** means p < 0.01 and *** means p < 0.001)., With the plot position serial number in the field as a random effect. CK: control; DP: decreased precipitation; IP: increased precipitation.

**Table S2.** Permutational multivariate analysis of variance using Bray-Curtis similarity values on bacterial community matrix in different precipitation levels

| Data | Factors | Sum of sqrs | df | Mean square | F | p |
| --- | --- | --- | --- | --- | --- | --- |
| Bacteria | Year | 0.54 | 2 | 0.27 | 2.83 | 0.001 |
|  | precipitation | 0.28 | 2 | 0.14 | 1.51 | 0.094 |
|  | Interaction | 0.39 | 3 | 0.096 | 1.01 | 0.437 |

**Table S3.** Proportion of relative dominant bacterial phylum in control, increased precipitation and decreased precipitation networks in 2016, 2018 and 2019.

|  | 2016 | | | 2018 | | | 2019 | | |
| --- | --- | --- | --- | --- | --- | --- | --- | --- | --- |
|  | CK | RD | RI | CK | RD | RI | CK | RD | RI |
| *Actinobacteriota* | 23.3% | 25.4% | 24.7% | 24.2% | 25.4% | 24.9% | 25.2% | 24.8% | 25.0% |
| *Acidobacteriota* | 20.0% | 16.8% | 17.5% | 18.1% | 19.0% | 176% | 18.1% | 18.1% | 18.4% |
| *Chloroflexi* | 17.2% | 17.7% | 18.0% | 17.2% | 18.1% | 17.6% | 18.1% | 17.7% | 17.1% |
| *Proteobacteria* | 13.9% | 14.8% | 14.8% | 14.9% | 14.0% | 13.7% | 12.8% | 12.8% | 14.5% |
| *Gemmatimonadota* | 7.4% | 8.6% | 8.5% | 8.8% | 7.7% | 8.2% | 8.0% | 8.0% | 7.5% |
| *Myxococcota* | 7.4% | 6.2% | 6.7% | 7.05% | 6.8% | 7.3% | 7.5% | 8.0% | 7.0% |
| *Firmicutes* | 2.8% | 2.9% | 2.7% | 4.0% | 3.17% | 3.9% | 3.5% | 3.5% | 4.0% |
| *Cyanobacteria* | 1.4% | 1.4% | 0.9% | 0.8% | 0.9% | 1.3% | 1.3% | 0.4% | 1.3% |
| *Planctomycetota* | 1.4% | 1.4% | 1.3% | 1.3% | 1.4% | 1.3% | 0.9% | 1.3% | 0.4% |

CK: control; DP: decreased precipitation; IP: increased precipitation.

**Table S4.** The keystone taxonomic composition in different precipitation gradient in 2016, 2018 and 2019

| Year | Treatment | Phylum | Pi-value | Zi-value | Keystone classification |
| --- | --- | --- | --- | --- | --- |
| 2016 | IP | Chloroflexi | 0.67 | -1.6 | connectors |
| 2018 | CK | Gemmatimonadota | 0.63 | -0.35 | connectors |
| 2018 | CK | Chloroflexi | 0.64 | -0.91 | connectors |
| 2018 | CK | Actinobacteriota | 0.67 | -1.32 | connectors |
| 2018 | DP | Firmicutes | 0 | 2.6 | Module hubs |
| 2019 | CK | Acidobacteriota | 0.63 | -0.61 | connectors |
| 2019 | CK | Actinobacteriota | 0.62 | 0.51 | connectors |
| 2019 | CK | Actinobacteriota | 0.64 | 0.82 | connectors |
| 2019 | CK | Actinobacteriota | 0.62 | 1.17 | connectors |
| 2019 | CK | Actinobacteriota | 0.62 | 1.17 | connectors |
| 2019 | IP | Actinobacteriota | 0.63 | 0 | connectors |
| 2019 | IP | Actinobacteriota | 0.64 | 0 | connectors |
| 2019 | IP | Chloroflexi | 0.62 | -1.5 | connectors |
| 2019 | IP | Chloroflexi | 0.63 | 0 | connectors |
| 2019 | IP | Proteobacteria | 0.63 | 0 | connectors |
| 2019 | IP | Chloroflexi | 0 | 3.06 | Module hubs |
| 2019 | IP | Myxococcota | 0 | 2.64 | Module hubs |

CK: control; DP: decreased precipitation; IP: increased precipitation.

|  |  | pH | EC | TN (mg g−1) | TP (mg g−1) | TC (mg g−1) | SM | AgB | BgB(g/m^2^) |
| --- | --- | --- | --- | --- | --- | --- | --- | --- | --- |
| 2016 | CK | 8.63±0.13 | 137.73±18.15 | 0.69±0.03 | 0.16±0 | 9.88±0.54 | 11.29±0.1b | 272.06±4.29b | 201.68±9.88 |
|  | DP | 8.43±0.03 | 119.2±6.61 | 0.71±0.01 | 0.15±0.01 | 8.92±0.11 | 9.68±0.17c | 249.89±7.02c1 | 201.74±11.93 |
|  | IP | 8.69±0.17 | 139.08±22.86 | 0.66±0.03 | 0.15±0.01 | 8.97±0.24 | 12.94±0.23a | 383±15.49a | 269.14±26.29 |
|  |  |  |  |  |  |  |  |  |  |
| 2018 | CK | 9.1±0.23 | 394.67±59.24 | 0.76±0.04b | 0.21±0.04 | 6.98±0.29b | 10.44±0.1b | 154.22±5.53a | 177.03±18.78 |
|  | DP | 8.98±0.27 | 372.17±62.8 | 0.81±0.05b | 0.15±0.02 | 7.66±0.49b | 8.67±0.06c | 119.29±8.44b | 159.13±14.91 |
|  | IP | 8.42±0.26 | 249.93±52.6 | 0.99±0.05a | 0.2±0.02 | 9.45±0.6a | 12.09±0.15a | 165.23±3.67a | 230.28±35.64 |
|  |  |  |  |  |  |  |  |  |  |
| 2019 | CK | 9.08±0.22a | 252.5±18.58b | 0.80±0.06b | 0.13±0.01b | 7.52±0.58b | 12.14±0.13b | 161.45±1.21b | 211.38±38.29b |
|  | DP | 8.93±0.23a | 261±9.61a | 0.79±0.04b | 0.19±0.03ab | 7.17±0.27b | 10.69±0.15c | 148.95±5.36b | 153.35±19.64b |
|  | IP | 8.23±0.20b | 168.3±26.46c | 0.97±0.05a | 0.22±0.02a | 9.66±0.6a | 13.67±0.22a | 212.22±12.11a | 384.39±16.92a |

Table S5. Soil properties in increased or decreased precipitation in 2016, 2018 and 2019. Significant differences between different precipitation levels are indicated by lowercase letters.

One-way ANOVA was used to test difference significance among treatments, only significant differences between treatments was labeled with letter. CK: control; DP: decreased precipitation; IP: increased precipitation. EC: electrical conductivity; TC: soil total carbon; TN: soil total nitrogen; TP: soil total phosphorus; SM: soil moisture; AgB: aboveground biomass; BgB: belowground biomass. values are means ± 1 SE (n = 6).
